# P-382. Evaluation of Antibiotic Prophylaxis for Patients Undergoing Whipple Procedure

**DOI:** 10.1093/ofid/ofae631.583

**Published:** 2025-01-29

**Authors:** Garrett Crawford, Caitlin Soto, Y Vivian Tsai, Sara E Cosgrove, Eili Klein, Kate Dzintars

**Affiliations:** The Johns Hopkins Hospital, Baltimore, Maryland; The Johns Hopkins Hosiptal, Baltimore, Maryland; The Johns Hopkins Hospital, Baltimore, Maryland; Johns Hopkins School of Medicine, Baltimore, MD; Johns Hopkins School of Medicine, Baltimore, MD; The Johns Hopkins Hospital, Baltimore, Maryland

## Abstract

**Background:**

Pancreatoduodenectomy (PD) or the Whipple is a complex surgical intervention with high complication rates. The purpose of this study is to evaluate the efficacy of standard of care (SOC) perioperative antibiotics, cefazolin plus metronidazole, as prophylaxis for surgical site infection (SSI) in patients undergoing PD at The Johns Hopkins Hospital (JHH).

**Methods:**

A retrospective study was performed of adult patients undergoing PD at JHH between January 1, 2022 and October 31, 2023. The following information was collected on all patients: peri-operative antibiotics administered, placement of operative drains, pre-operative biliary stent, prior endoscopic retrograde cholangiopancreatography, SSI, pancreatic fistula, and microbiological data. Criteria for SSIs were adapted from the CDC’s National Healthcare Safety Network definitions. SOC prophylaxis included cefazolin plus metronidazole. The primary outcome was the proportion of patients who developed an SSI within 30 days of PD. Secondary outcomes included identification of the causative pathogens of SSI in patients after PD and the proportion of organisms susceptible to SOC antibiotics. Descriptive statistics was used to summarize demographic data and the primary and secondary outcomes.

**Results:**

A total of 333 patients met inclusion criteria and were documented as having PD during our study period. Of these patients, 106 (31.2%) had post-operative complications including infection or fistula. Overall, 81 patients (24.3%) had a SSI after PD. Seventy-one SSIs occurred in 295 patients (24.1%) receiving SOC antibiotic prophylaxis compared to 10 SSIs in 38 patients (26.3%) receiving non-SOC antibiotics (p=0.761). Of the 81 patients with a SSI, pathogens were identified through post-operative culture data in 33 patients. Thirty-two of the 55 (58%) clinically relevant pathogens were susceptible to SOC antibiotics.

**Conclusion:**

The incidence of surgical complications associated with PD at our institution is comparable to rates reported in the available literature. While the incidence of clinically relevant organisms not covered by SOC prophylaxis is low, a targeted approach for prophylaxis may be required for select patients.

**Disclosures:**

**All Authors**: No reported disclosures

